# Housing Temperature Influences Atypical Antipsychotic Drug‐Induced Bone Loss in Female C57BL/6J Mice

**DOI:** 10.1002/jbm4.10541

**Published:** 2021-09-07

**Authors:** Roni F Kunst, Audrie L Langlais, Deborah Barlow, Karen L Houseknecht, Katherine J Motyl

**Affiliations:** ^1^ Center for Molecular Medicine Maine Medical Center Research Institute Scarborough ME USA; ^2^ Graduate School of Biomedical Sciences and Engineering, University of Maine Orono ME USA; ^3^ College of Osteopathic Medicine, University of New England Biddeford ME USA; ^4^ Tufts University School of Medicine, Tufts University Boston MA USA

**Keywords:** ANIMAL MODELS, PRECLINICAL STUDIES, SYSTEMS BIOLOGY—BONE INTERACTORS, BONE‐BRAIN‐NERVOUS SYSTEM INTERACTIONS, SYSTEMS BIOLOGY—BONE INTERACTORS, BONE‐FAT INTERACTIONS

## Abstract

Atypical antipsychotic (AA) drugs, such as risperidone, are associated with endocrine and metabolic side effects, including impaired bone mineral density (BMD) acquisition and increased fracture risk. We have previously shown that risperidone causes bone loss through the sympathetic nervous system and that bone loss is associated with elevated markers of thermogenesis in brown and white adipose tissue. Because rodents are normally housed in sub‐thermoneutral conditions, we wanted to test whether increasing housing temperature would protect against bone loss from risperidone. Four weeks of risperidone treatment in female C57BL/6J mice at thermoneutral (28°C) housing attenuated risperidone‐induced trabecular bone loss and led to a low‐turnover bone phenotype, with indices of both bone formation and resorption suppressed in mice with risperidone treatment at thermoneutrality, whereas indices of bone resorption were elevated by risperidone at room temperature. Protection against trabecular bone loss was not absolute, however, and additional evidence of cortical bone loss emerged in risperidone‐treated mice at thermoneutrality. Taken together, these findings suggest thermal challenge may be in part responsible for bone loss with risperidone treatment and that housing temperature should be considered when assessing bone outcomes of treatments that impact thermogenic pathways. © 2021 The Authors. *JBMR Plus* published by Wiley Periodicals LLC on behalf of American Society for Bone and Mineral Research.

## Graphical abstract

Room‐temperature housing (left panel, 23°C) mounts a thermogenic stress response (large spark) to maintain body temperature. The atypical antipsychotic (AA) risperidone (RIS) also induces thermogenesis, accompanied by trabecular bone loss. Increasing housing temperature (right panel, 28°C) rescues RIS‐induced trabecular bone loss. Findings support an understudied thermogenic mechanism of bone loss side effects from AA drugs. Created with BioRender.com.

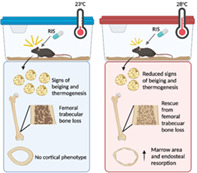

## Introduction

Atypical antipsychotic (AA) drugs (a.k.a. second‐generation antipsychotic drugs) are used to treat mental disorders such as schizophrenia and bipolar disorder. However, AA drugs are also prescribed off‐label for other conditions, at a range of ages from adolescence to elderly, despite evidence of hormonal and metabolic side effects and “black box” warnings of increased risk of death due to cardiac arrest.^(^ ^1^ ^)^ Furthermore, elevated fracture risk has been increasingly recognized as a side effect of AA drugs. AA drugs can cause hyperprolactinemia due to their affinity for the dopamine receptor. High prolactin, in turn, downregulates the production of sex steroids, which increases susceptibility to impaired bone gain, osteoporosis, and fracture.^(^ ^2^, ^3^, ^4^ ^)^ Although this mechanism of bone loss undoubtedly occurs in some patients treated with AA drugs, additional mechanisms beyond hypogonadism are likely because not all patients present with hyperprolactinemia, and postmenopausal women are also susceptible to elevated fracture risk from AA drugs. Consistent with this, ovariectomized mice lose additional bone in response to the AA drug risperidone (RIS), indicating that hypogonadism is not the only contributing factor.^(^ ^5^ ^)^

Other studies suggest that AA drugs cause bone loss by elevating sympathetic nervous system (SNS) activity. The β2AR is expressed in bone, and elevated SNS activity has been shown to cause bone loss by reducing bone formation and increasing resorption.^(^ ^6^, ^7^ ^)^ Although AA drugs do not bind βARs, co‐treatment of mice with the AA drug risperidone and the non‐specific βAR‐antagonist (β‐blocker) propranolol attenuated risperidone‐induced bone loss.^(^ ^8^ ^)^ Furthermore, risperidone did not have an effect on bone in mice null for *Adrb2*, the gene encoding β2AR, supporting the role of the SNS in AA‐induced bone loss.^(^ ^8^ ^)^ Selective serotonin reuptake inhibitors (SSRIs) also cause bone loss by desensitizing serotonin signaling and subsequently increasing SNS tone,^(^ ^9^ ^)^ and risperidone action as a serotonin receptor antagonist may work in a similar way. Despite this, the cause of elevated SNS activity after AA drug treatment is unclear and may be centrally mediated through direct effects on CNS centers responsible for SNS outflow or related to the physiological responses to AA drug exposure.^(^ ^10^ ^)^ One such response, in particular, is the finding that preclinical animal models have increased markers of thermogenesis in brown adipose tissue after treatment with AA drugs.^(^ ^8^, ^11^ ^)^ Several studies have linked the control of thermogenesis to bone turnover.^(^ ^12^, ^13^, ^14^, ^15^, ^16^, ^17^ ^)^ However, heightened thermogenesis is generally associated with fat mass loss, which would be contradictory to the clinical findings of weight gain and metabolic dysfunction in patients treated with AA drugs.

Unlike room temperature (~20 to 25°C) for humans, temperatures of ~26 to 34°C are considered thermoneutral for rodents.^(^ ^18^, ^19^ ^)^ The thermoneutral (TN) temperature of a species is defined as the temperature where endotherms function at their basal metabolic rate while maintaining their core body temperature.^(^ ^19^ ^)^ Thus, room temperature (RT) is below rodent thermoneutrality, requiring increased heat production through non‐shivering thermogenesis to counterbalance heat loss. It is conceivable, therefore, that since rodent studies conducted with AA drugs were previously performed at RT, mice are already in a heightened state of thermogenesis, cannot gain weight on AA drugs, and may present with signs of cold stress and subsequent bone loss upon exposure to an AA drug. Animal studies have shown that low environmental temperatures cause trabecular bone loss, which may be due at least in part to elevated SNS activity.^(^ ^15^, ^17^ ^)^ However, whether bone loss at sub‐thermoneutral conditions is due solely to the SNS remains unclear because the non‐selective β‐blocker propranolol only partially protected against bone loss below thermoneutrality.^(^ ^16^ ^)^ Although the mechanisms of housing temperature effects on bone remain unclear, it is important to test how models of bone loss, that simultaneously affect thermoregulatory functions, perform at different housing temperature conditions.

Collectively, these findings led us to hypothesize that rodent housing temperature may influence the physiological responses to AA drugs, including bone loss. In the current study, we administered vehicle or risperidone to 8‐week‐old C57BL/6J female mice housed at either RT or TN temperature. We successfully suppressed RIS‐induced markers of beige and brown adipose tissue induction at TN temperatures, as well as attenuated trabecular bone loss from risperidone. Our results further support the bone‐protective effects of TN temperature in young wild‐type mice by suppressing bone turnover. However, there was some evidence that bone loss may be site‐ and temperature‐specific because TN housing protected against trabecular bone loss, but risperidone significantly affected cortical bone in mice housed at TN. Taken together, our findings stress the importance of considering rodent housing temperature when examining bone outcomes from pharmaceuticals that influence beige and brown adipose tissue.

## Materials and Methods

### Mice and treatment

Female C57BL/6J mice were used. We have established that risperidone induces bone loss in both male and female mice, but subsequent studies demonstrating the role of the SNS in risperidone‐induced bone loss were performed in female mice.^(^ ^8^, ^20^ ^)^ Mice were obtained from the Jackson Laboratory (Bar Harbor, ME, USA) at 7 weeks of age and randomly assigned to treatment groups. Mice were housed at room temperature (23°C) or thermoneutrality (28 to 30°C) using a rodent incubator (Power Scientific, Doylestown, PA, USA) on 14:10‐hour light/dark cycle and fed normal laboratory chow and water *ad libitum*. At 8 weeks of age, mice from each temperature were treated daily for 4 weeks with 1.5 mg/kg risperidone (*n* = 10 at RT, 10 at TN) or 0.1% acetic acid vehicle by oral gavage (*n* = 9 at RT, 10 at TN). The dose of risperidone was chosen based on our previous dose‐ranging pharmacokinetic studies that demonstrated oral gavage of 1.5 mg/kg risperidone resulted in clinically relevant serum concentrations.^(^ ^5^, ^10^ ^)^ The age of the mice was chosen because our past studies have shown that bone loss from risperidone was limited to the trabecular compartment, and 8 weeks is the age when trabecular bone reaches peak bone volume fraction (BV/TV).^(^ ^5^, ^8^, ^21^ ^)^ Cages were changed weekly and preheated for 24 hours before adding mice in the warm temperature incubator. To ensure there were no residual acute drug effects on the day of euthanization, mice received their last dose of drug (risperidone or vehicle) on day 26, the morning before euthanization. The following day (day 27), all mice were euthanized by decapitation after isoflurane anesthesia. All mouse procedures were approved by the Institutional Animal Care and Use Committee (IACUC) at Maine Medical Center Research Institute (MMCRI). Blood was collected and tissues for mRNA expression snap‐frozen in liquid nitrogen, while tissues for micro‐computed tomography (μCT) and histology were fixed in 10% neutral buffered formalin. After 48 hours, the 10% formalin solution was replaced by 70% ethanol.

### Dual‐energy X‐ray absorptiometry

At 8 and 12 weeks of age, before start of the treatment and at time of harvest, mice underwent dual‐energy X‐ray absorptiometry (DXA) by the PIXImus densitometer, calibrated with a mouse phantom provided by the manufacturer (GE Lunar, Madison, WI, USA). Mice were anesthetized by 2% isoflurane and placed ventral side down with each limb and tail positioned away from the body. Full‐body scans were obtained according to the manufacturer's protocol. The supplied software (Lunar PIXImus 2, version 2.1) was used to assess bone mineral density (BMD), bone mineral content (BMC), lean mass, and fat mass. The head was excluded from the full‐body scan because of its high mineral density in the skull and teeth.

### Micro‐computed tomography

The microarchitecture of the distal trabecular bone and midshaft cortical bone of the femur were analyzed by μCT (resolution 10.5 μm, VivaCT‐40, Scanco Medical AG, Bassersdorf, Switzerland). Image acquisition and analysis protocols adhered to guidelines for the assessment of rodent bones by μCT.^(^ ^22^ ^)^ In the femur, trabecular microarchitecture was evaluated in a 1575 μm (150 transverse slices) region beginning 200 μm superior to the peak of the growth plate and extending proximally. A fixed threshold of 350 mgHA/cm^3^ was used to separate bone from soft tissue. Measurements included femur bone volume/total volume (BV/TV), trabecular BMD, trabecular number (Tb.N), trabecular thickness (Tb.Th), trabecular separation (Tb.Sp), connectivity density (Conn.D), and structure model index (SMI). Cortical region scans at the midpoint of each femur were performed with an isotropic pixel size of 10.5 μm and slice thickness of 10.5 μm. Fifty consecutive slices (for a total length of 525 μm), with a fixed threshold of 700 mgHA/cm^3^, were used to segment cortical bone from tissue. The following variables were calculated: average cortical bone area (Ct.Ar), marrow area (M.Ar), total cross‐sectional area (T.Ar), cortical area fraction (Ct.Ar/T.Ar), cortical porosity (Ct.Porosity), and tissue mineral density (TMD). All scans were analyzed using the software of the manufacturer (Scanco Medical AG, version 4.05).

### Serum measurements

Blood was collected after decapitation. Serum was isolated after centrifugation and stored at −80°C. Serum CTX‐I and P1NP concentration was determined with, respectively, the RatLaps CTX‐I and Rat/Mouse P1NP enzyme immunoassays (EIA, Immunodiagnostic Systems, Boldon, UK) following the manufacturer's protocol. Samples were read on a FlexStation 3 plate reader (Molecular Devices, Eugene, OR, USA). Calculations were determined using FlexStation software with a 4‐parameter logistic curve as suggested in EIA manuals.

### Histology and marrow adipocyte quantification

Gonadal fat, inguinal fat, brown adipose tissue, and decalcified femurs were embedded in paraffin and cut to 4‐ to 5‐μm sections. Sections were stained with hematoxylin and eosin (H&E) in the MMCRI Histology Core using standard protocols.

Quantification of bone marrow adipocytes was done using Fiji (Image J). First, a global scale was set (1000 pixels/mm). The ROI was set 0.1 mm proximal to the distal growth plate and extending proximally to include the secondary spongiosa but excluding cortical bone. The total ROI area (mm^2^) was calculated and used to normalize adipocyte data. Next, images were converted to 32‐bit, and a threshold was set using the “Huang” filter. The filter was manually adjusted from the automatic setting as necessary to exclude trabecular bone or tears. Using Adjustable Watershed (range 0.3 to 0.7) and Despeckle, the threshold was optimized to resolve neighboring adipocytes. Finally, using the Analyze Particles tool, the number and area of individual adipocytes were calculated with the following settings: circumference 50 to 2500 pixels and circularity −0.06 to 1.00. The person performing the analysis was blinded to treatment groups and reviewed the results to exclude any misidentified adipocytes or manually measure adipocytes that were not properly included but still present within the ROI. This method was previously validated against standard methods of manual adipocyte quantification in female mice.^(^ ^23^ ^)^

### RNA isolation, cDNA formation, and RT‐qPCR

Tissues were snap‐frozen in liquid nitrogen and stored at −80°C. mRNA was isolated from tibia cortical shell and bone marrow and brown adipose tissue (BAT) under liquid nitrogen conditions. Samples were crushed by a chilled mortar and pestle and homogenized in 1 mL TriReagent (MRC, Cincinnati, OH, USA). Subsequently, 200 μL CHCl3 was added and after 15 minutes incubation, samples were centrifuged at 15,800 *g* at 4°C. The aqueous upper layer is transferred and mixed with 500 μL isopropanol before frozen overnight at −80°C. The next day, the pellet is washed twice in 75% EtOH and dissolved in fresh MilliQ water. RNA concentration was determined with the NanoDrop 2000 and 1000 ng RNA was entered in every cDNA reaction, together with 10× RT buffer, 25× dNTP, 10× random primers, reverse transcriptase, and nuclease‐free H_2_O. After the PCR program, which consisted of 10 minutes at 25°C, 120 minutes at 37°C, 5 minutes at 85°C, and cooling to 4°C, samples were directly diluted with 180 μL MilliQ. Three microliters of each cDNA sample were then used for the RT‐qPCR analysis, together with a mixture of water, SYBR green (BioRad, Hercules, CA, USA), and forward and reverse primers from IDT (Coralville, IA, USA) or Primer Design (Southampton, UK) (Supplemental Table S1).

### Statistics

Statistical significance was determined by two‐way ANOVA, with drug treatment and housing temperature as independent variables. Only after a significant interaction effect, we performed a Tukey's *post hoc* test to determine pairwise significance. ANOVA was performed using Graph Pad Prism 9 (GraphPad, La Jolla, CA, USA).

## Results

Four weeks of risperidone treatment at either room temperature (23°C) or thermoneutrality (28 to 30°C) significantly increased body mass, regardless of housing temperature (Supplemental Table S2). This was largely due to a significant RIS‐induced increase in lean mass; however, the increase in lean mass from baseline was suppressed by thermoneutral housing. RIS did not influence fat mass, but as expected, TN housing significantly increased fat mass in both vehicle‐ and RIS‐treated mice (Supplemental Table S2). TN housing increased endpoint whole‐body aBMD, aBMC, and femoral aBMD (Supplemental Table S3). However, percent change in these parameters was not significantly altered by TN housing, suggesting differences were subtle and potentially confounded by differences at baseline. Risperidone, on the other hand, did not significantly alter endpoint or % change in bone parameters by DXA, which is consistent with our previous studies and the limitations in the sensitivity of DXA for detecting trabecular bone changes.^(^ ^5^, ^8^ ^)^

**Fig 1:**
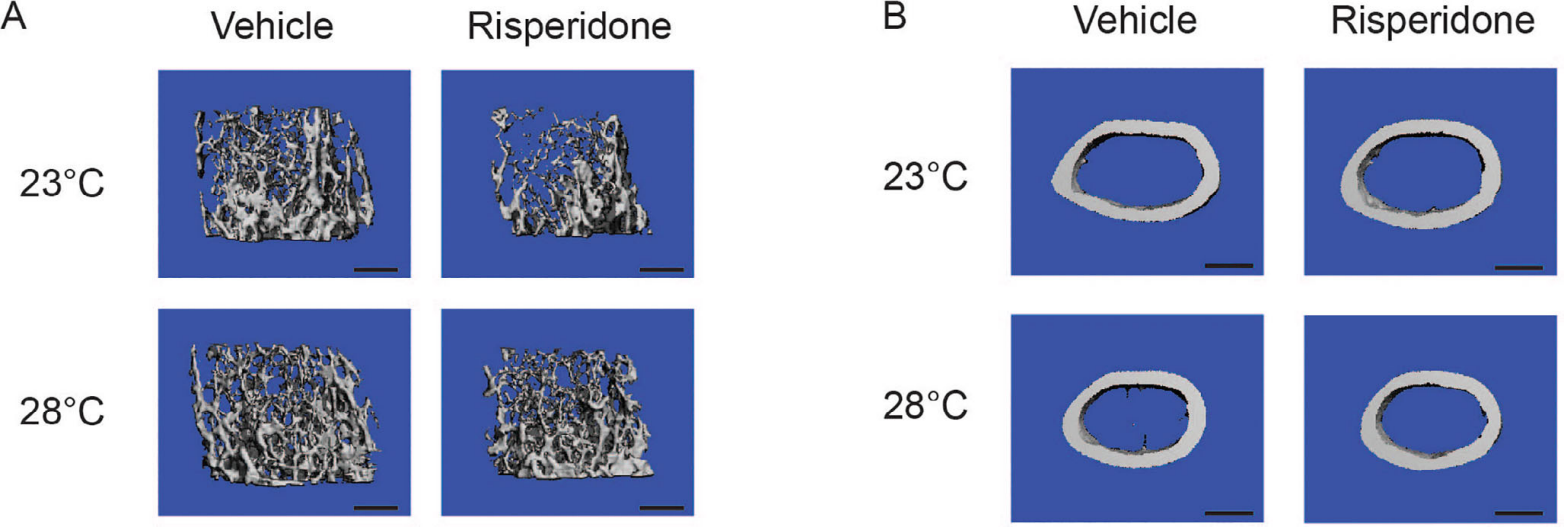
Trabecular bone loss from risperidone is attenuated in warmer housing. Mice were housed at room temperature (23°C) or thermoneutral temperature (28°C) for 4 weeks with 1.5 mg/kg risperidone or vehicle (0.1% acetic acid). (*A*) Representative μCT images of trabecular bone at the distal metaphysis. (*B*) Representative μCT images of midshaft cortical bone. Scale bar = 500 μm.

RIS significantly decreased trabecular BV/TV, BMD, Conn.D, and Tb.N but increased Tb.Th and Tb.Sp compared with vehicle (Fig. 1*A*; Table 1). {FIG1}{TBL 1} Housing mice at TN increased BV/TV, BMD, Conn.D, SMI, and Tb.N and reduced Tb.Sp. Interaction effects tended toward significance with Conn.D and Tb.N and were significant with regard to Tb.Sp. Specifically, the RIS‐induced increase in Tb.Sp was blunted in TN compared with RT (22.46% increase at RT versus 12.32% increase at TN). BMD loss with RIS at RT was 27% compared with only 12% at TN. Overall, these findings suggest that TN housing did not completely protect mice from RIS‐induced trabecular bone loss but did diminish the severity of bone loss.

**Table 1:** Trabecular Microarchitecture of the Distal Femur of Female Mice Treated With Risperidone or Vehicle, at Room Temperature or Thermoneutrality, for 4 Weeks

Trabecular bone	Room temperature		Thermoneutral		2‐Way ANOVA *p* value			% Change RIS:VEH	
	Veh (*n* = 9)	RIS (*n* = 9)	Veh (*n* = 10)	RIS (*n* = 10)	Drug	Temp	Int.	RT	TN
BV/TV (%)	9.5 ± 0.3	7.6 ± 0.3	12.3 ± 0.3	10.9 ± 0.6	**0.0002**	**<0.0001**	0.5933	−19.1	−11.4
BMD (mg/cm^3^)	80.1 ± 4.3	58.3 ± 4.2	108.0 ± 2.3	95.6 ± 5.9	**0.0004**	**<0.0001**	0.2905	−27.2	−11.5
Conn.D (1/mm^3^)	81.9 ± 4.1	54.9 ± 3.5	130.6 ± 5.6	86.1 ± 5.7	**<0.0001**	**<0.0001**	0.0856	−33.0	−34.1
SMI	2.90 ± 0.05	2.94 ± 0.06	2.55 ± 0.05	2.65 ± 0.06	0.2286	**<0.0001**	0.5532	1.2	4.0
Tb.N (1/mm)	4.22 ± 0.06	3.50 ± 0.08	4.64 ± 0.04	4.20 ± 0.10	**<0.0001**	**<0.0001**	0.0624	−17.1	−9.5
Tb.Th (mm)	0.0454 ± 0.0003	0.0480 ± 0.0007	0.0465 ± 0.0005	0.0482 ± 0.0007	**0.0009**	0.2925	0.5054	5.6	3.8
Tb.Sp (mm)	0.236 ± 0.004	0.289 ± 0.007****	0.212 ± 0.002*	0.238 ± 0.006**,****	**<0.0001**	**<0.0001**	**0.0124**	22.5	12.3

Data presented as mean ± SEM.

Veh = vehicle; RIS = risperidone; RT = room temperature; TN = thermoneutral; BV/TV = bone volume/total volume; BMD = bone mineral density; Conn.D = connectivity density; SMI = structure model index; Tb.N = trabecular number; Tb.Th = trabecular thickness; Tb.Sp = trabecular separation.

2‐way ANOVA main effects: bold values indicate significance with *p* < 0.05.

Pairwise comparisons: *p < 0.05 and **p < 0.005 compared with drug‐matched, room temperature–housed mice. ****p < 0.005 compared with housing‐matched, vehicle‐treated mice.

Compared with trabecular bone, cortical bone was largely unaffected by RIS treatment at RT (Fig. 1*B*; Table 2), {TBL 2} which is consistent with our previous work.^(^ ^5^, ^8^ ^)^ However, TN housing significantly increased Ct.Ar, bone area fraction (BA/TA), TMD, and Ct.Th, while significantly decreasing Ct.Por. Interaction terms were significant for medullary area (Ma.Ar), BA/TA, and Ct.Th, and post hoc test indicated that RIS caused significant BA/TA loss at TN temperatures only. This was likely due to increased medullary area (Ma.Ar, *p* = 0.051, compared with vehicle at TN). Therefore, RIS appears to reduce the positive effects of TN housing on cortical bone (Table 2).

**Table 2:** Cortical Microarchitecture of the Femur Midshaft of Female Mice Treated With Risperidone or Vehicle, at Room Temperature or Thermoneutrality, for 4 Weeks

Cortical bone	Room temperature		Thermoneutral		2‐Way ANOVA *p* value			% change RIS:VEH	
	Veh (*n* = 9)	RIS (*n* = 9)	Veh (*n* = 10)	RIS (*n* = 10)	Drug	Temp.	Int.	RT	TN
Ct.Ar (mm^2^)	0.68 ± 0.01	0.68 ± 0.01	0.72 ± 0.01	0.70 ± 0.01	0.5111	**0.0191**	0.3863	0.4	−2.9
Tt.Ar (mm^2^)	1.74 ± 0.02	1.72 ± 0.02	1.73 ± 0.02	1.77 ± 0.03	0.6834	0.4646	0.2153	−1.2	2.3
Ma.Ar (mm^2^)	1.06 ± 0.01	1.04 ± 0.02	1.00 ± 0.01	1.06 ± 0.02	0.2536	0.3440	**0.0138**	−2.2	6.1
BA/TA (%)	39.1 ± 0.5	39.7 ± 0.6	42.0 ± 0.5**	39.8 ± 0.4***	0.1530	**0.0050**	**0.0092**	1.7	−5.1
TMD (mg/cm^3^)	1101 ± 6	1107 ± 6	1142 ± 5	1130 ± 3	0.1070	**<0.0001**	0.5854	−0.6	−1.1
Ct.Por (%)	0.82 ± 0.05	0.89 ± 0.04	0.72 ± 0.03	0.76 ± 0.02	0.1572	**0.0040**	0.7241	8.1	5.6
Ct.Th (mm)	0.154 ± 0.002	0.157 ± 0.003	0.167 ± 0.002**	0.160 ± 0.002	0.3238	**0.0014**	**0.0425**	1.6	−4.2
pMOI (mm^4^)	0.320 ± 0.01	0.314 ± 0.001	0.332 ± 0.01	0.334 ± 0.01	0.8287	0.1501	0.7374	−1.8	0.4

Data presented as mean ± SEM.

Veh = vehicle; RIS = risperidone; RT = room temperature; TN = thermoneutral; Ct. = cortical; Ar = area; Tt. = total; Ma. = medullary; BA/TA = cortical bone fraction; TMD = tissue mineral density; Ct.Por = cortical porosity; Ct.Th = cortical thickness; pMOI = polar moment of inertia.

2‐way ANOVA main effects: bold values indicate significance with *p* < 0.05.

Pairwise comparisons: **p < 0.005 compared with drug‐matched, room temperature–housed mice. ***p < 0.05 compared with housing‐matched, vehicle‐treated mice.

**Fig 2:**
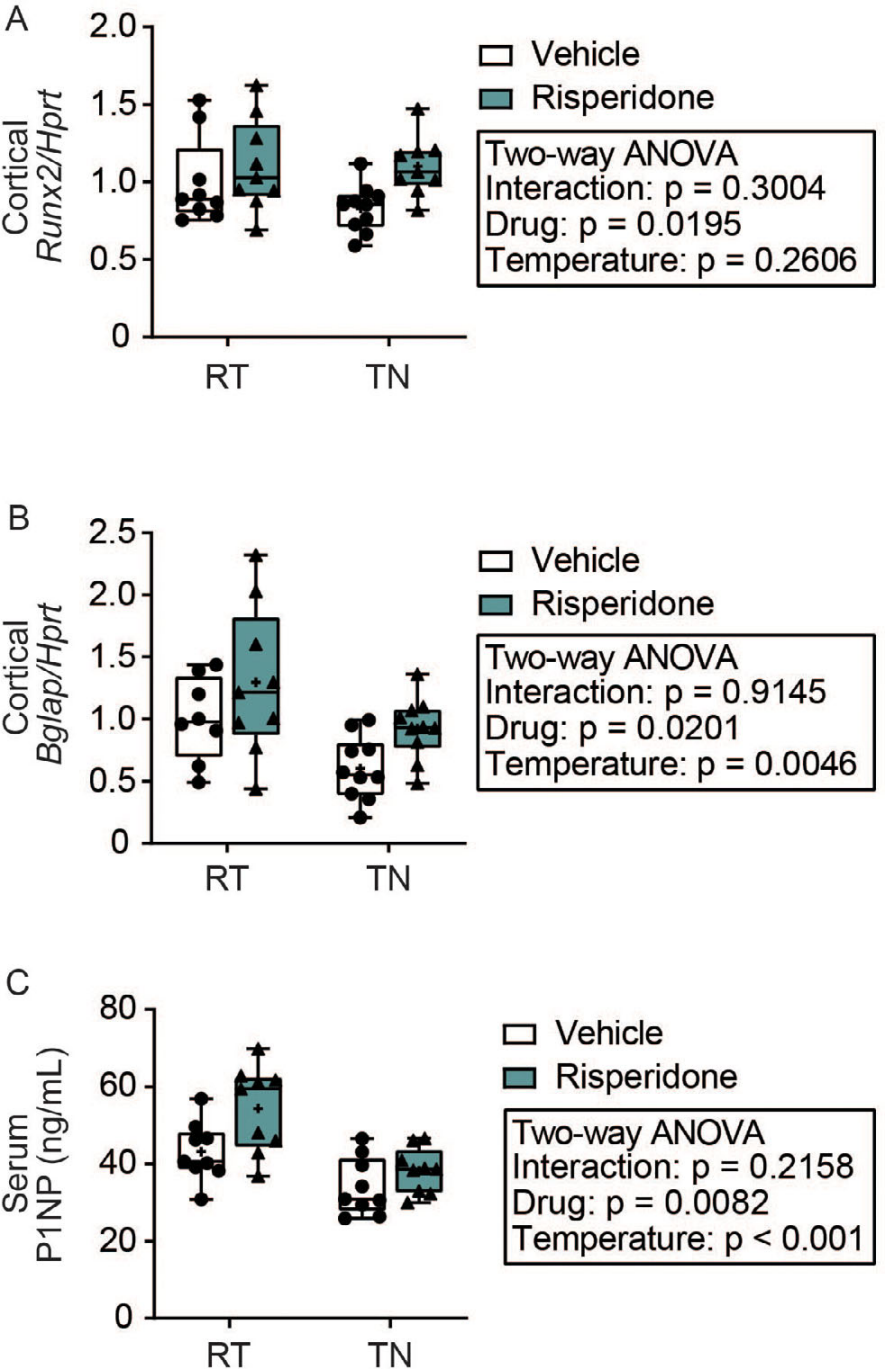
Risperidone increases and thermoneutral housing suppresses osteoblast activity markers. Mice were housed at room temperature (23°C) or thermoneutral (TN) temperature (28°C) for 4 weeks with 1.5 mg/kg risperidone or vehicle (0.1% acetic acid). (*A*) Gene expression of osteoblast differentiation transcription factor *Runx2* and (*B*) osteoblast marker *Bglap* (osteocalcin) normalized to *Hprt* expression from tibia cortical shell. *n* = 8–10 group. (*C*) Serum concentration of bone formation marker PINP determined by ELISA, *n* = 9–10 per group. Plots represent median (horizontal line) ± 95% confidence interval (wide bars), minimum and maximum (T bars) values, and 2‐way ANOVA results with Tukey's multiple comparisons test performed only after a significant (*p* < 0.05) interaction term. + = arithmetic mean.

To identify the etiology of bone changes, we examined osteoblast differentiation and bone formation markers, followed by marrow adiposity and osteoclast markers. RIS significantly increased *Runx2* and *Bglap* (gene encoding osteocalcin) expression in cortical shell independent of housing temperature (Fig. 2*A*, *B*). {FIG2} Serum procollagen type 1 N‐terminal pro‐peptide concentration (P1NP) was also significantly increased by RIS, suggesting bone formation may have been elevated despite RIS‐induced bone loss (Fig. 2*C*). However, both *Bglap* expression and serum P1NP were reduced by TN housing and none of the interaction terms for bone formation markers were significant.

**Fig 3:**
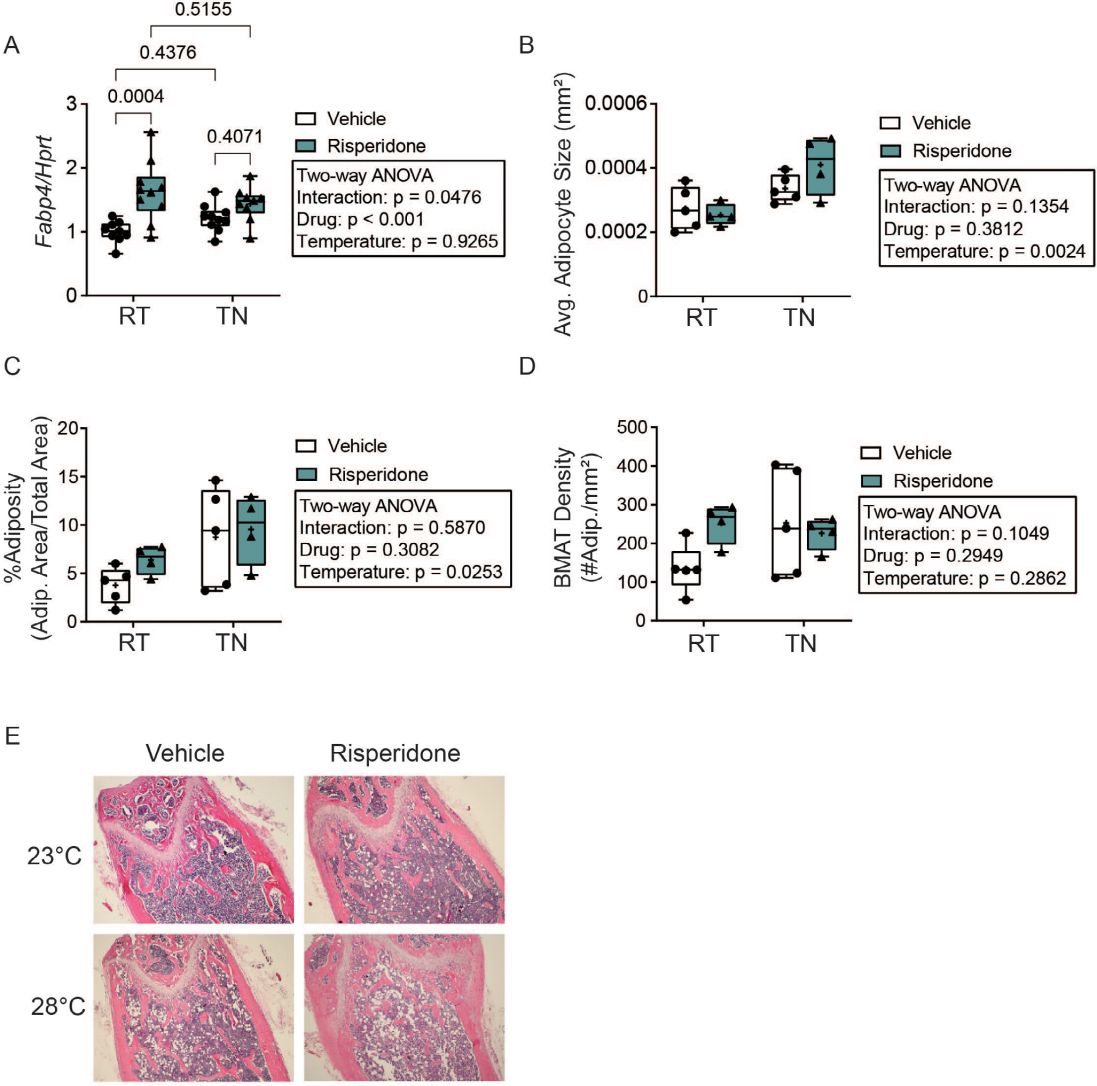
Thermoneutral housing–increased marrow adiposity is not affected by risperidone. (*A*) Gene expression of *Fabp4* normalized to *Hprt* expression from tibia marrow, *n* = 9–10 per group. (*B*–*D*) Image J quantification of marrow adipocytes. (*B*) Average adipocyte size, (*C*) adipocyte area/total area, (*D*) marrow adipocyte density at the distal metaphysis of the femur, *n* = 4–5 per group. Plots represent median (horizontal line) ± 95% confidence interval (wide bars), minimum and maximum (T bars) values, and 2‐way ANOVA results with Tukey's multiple comparisons test performed only after a significant (*p* < 0.05) interaction term. + = arithmetic mean. (*E*) Representative 4× images of H&E‐stained femur showing white adipocyte “ghosts” at the distal metaphysis.

Because osteoblasts and marrow adipocytes share a common progenitor, we next examined the effects of RIS and TN on marrow adiposity. RIS significantly increased the adipocyte marker *Fabp4* gene expression (Fig. 3*A*). {FIG3} However, this increase was blunted by housing mice at TN. To determine if this marker was indicative of quantitative changes in the marrow adipocytes, we analyzed adipocyte content of the distal femur metaphysis. RIS did not have any significant main effects on the average area (size) of individual adipocytes, adiposity (% area of marrow occupied by adipocytes), or adipose density (# adipocytes/mm^2^) (Fig. 3*B*–*E*). Thermoneutral housing increased adipocyte size and % adiposity but not marrow adipose density, and no histological parameters had significant interaction terms. Taken together, elevated bone formation markers and an absence of RIS‐induced changes in marrow adipose tissue suggest osteoblast‐adipocyte lineage selection is not influenced by RIS. However, suppression of bone formation markers at TN, coupled to elevated marrow adipose tissue, suggests housing temperature may play a role in osteoblast‐adipocyte lineage selection (Figs. 2 and 3).

**Fig 4:**
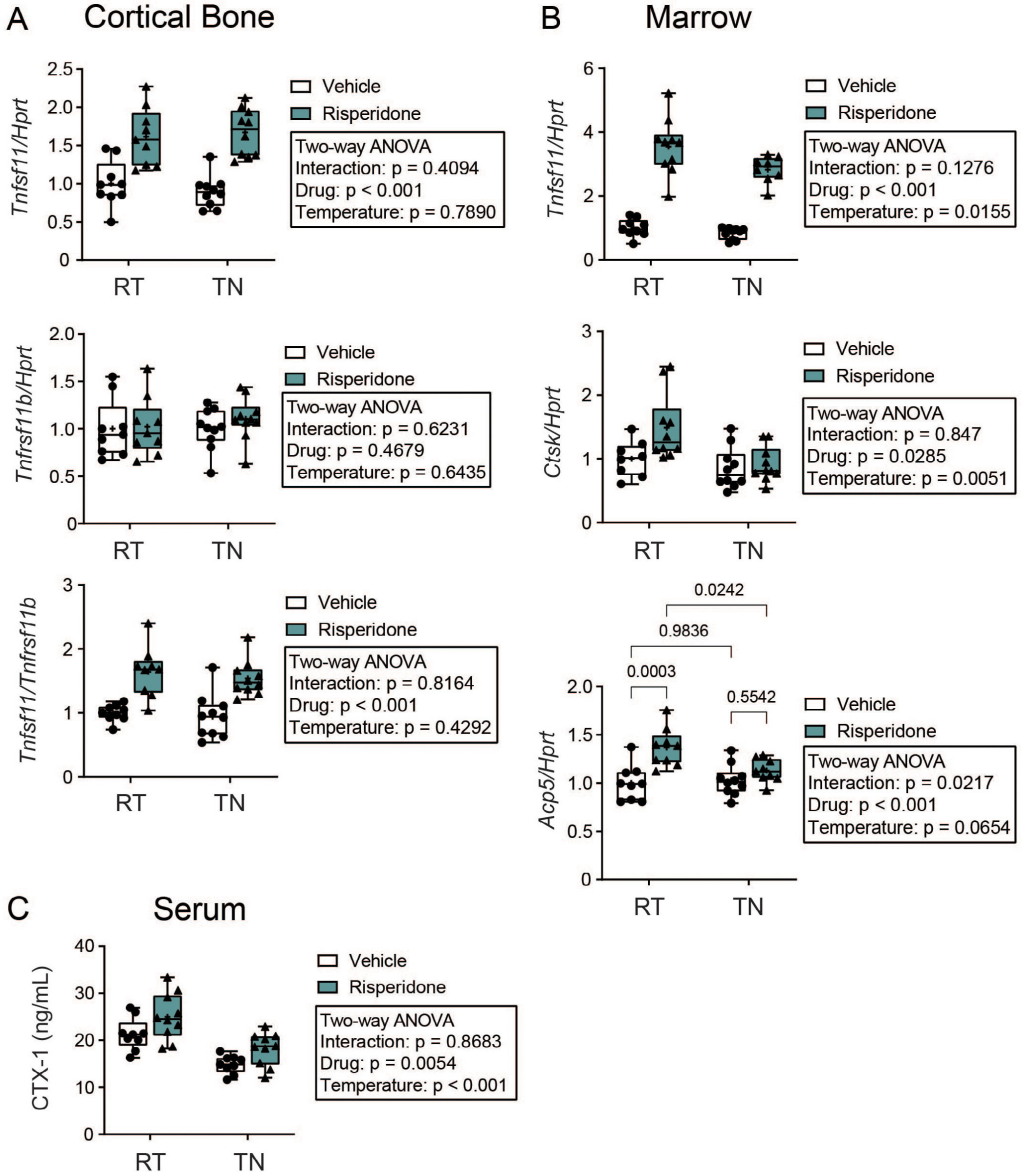
Thermoneutral housing suppresses risperidone‐induced osteoclast activity markers independent of RANKL. (*A*) Gene expression from tibia cortical bone of *Tnfsf11* (RankL) and *Tnfrsf11b* (Opg) normalized to *Hprt* and *Tnfsf11* normalized to *Tnfrsf11b*. (*B*) Gene expression from tibia marrow of *Tnfrsf11b* and osteoclast markers *Ctsk* and *Acp5* normalized to *Hprt*. *n* = 9–10 per group. (*C*) Serum concentration of bone resorption marker CTX‐1 determined by ELISA. *n* = 9–10 per group. Plots represent median (horizontal line) ± 95% confidence interval (wide bars), minimum and maximum (T bars) values, and 2‐way ANOVA results with Tukey's multiple comparisons test performed only after a significant (*p* < 0.05) interaction term. + = arithmetic mean.

Because bone formation markers could not explain microarchitectural changes in trabecular and cortical bone, we next examined osteoclast markers to see if increased resorption was coupled to increased formation. In tibia marrow and cortical shell, RIS significantly increased expression of *Tnfsf11* (gene encoding receptor activator of NF‐κB ligand [RANKL]), without changing *Tnfrsf11b* (gene encoding RANKL decoy receptor osteoprotegerin [OPG]), suggesting increased osteoclastogenesis (Fig. 4*A*, *B*). {FIG4} Consistently, mature osteoclast genes *Acp5* and *Ctsk* were also significantly increased by RIS in marrow (Fig. 4*B*). Serum levels of C‐terminal telopeptide‐1 (CTX‐1) were significantly increased by RIS regardless of temperature, further supporting that RIS increases osteoclast differentiation and resorption (Fig. 3*C*). Interestingly, housing temperature did not influence cortical bone *Tnfsf11*, but TN housing suppressed marrow *Tnfsf11* and *Ctsk* expression, as well as serum CTX‐1. In these parameters, the magnitude of the RIS‐induced increase was lower at TN than at RT. Furthermore, there was a significant interaction effect on marrow *Acp5* expression, such that RIS was not able to induce *Acp5* at TN temperature. Taken together, bone resorption markers suggest suppressed osteoclast activity at TN conditions may account for diminished severity of bone loss from RIS.

**Fig 5:**
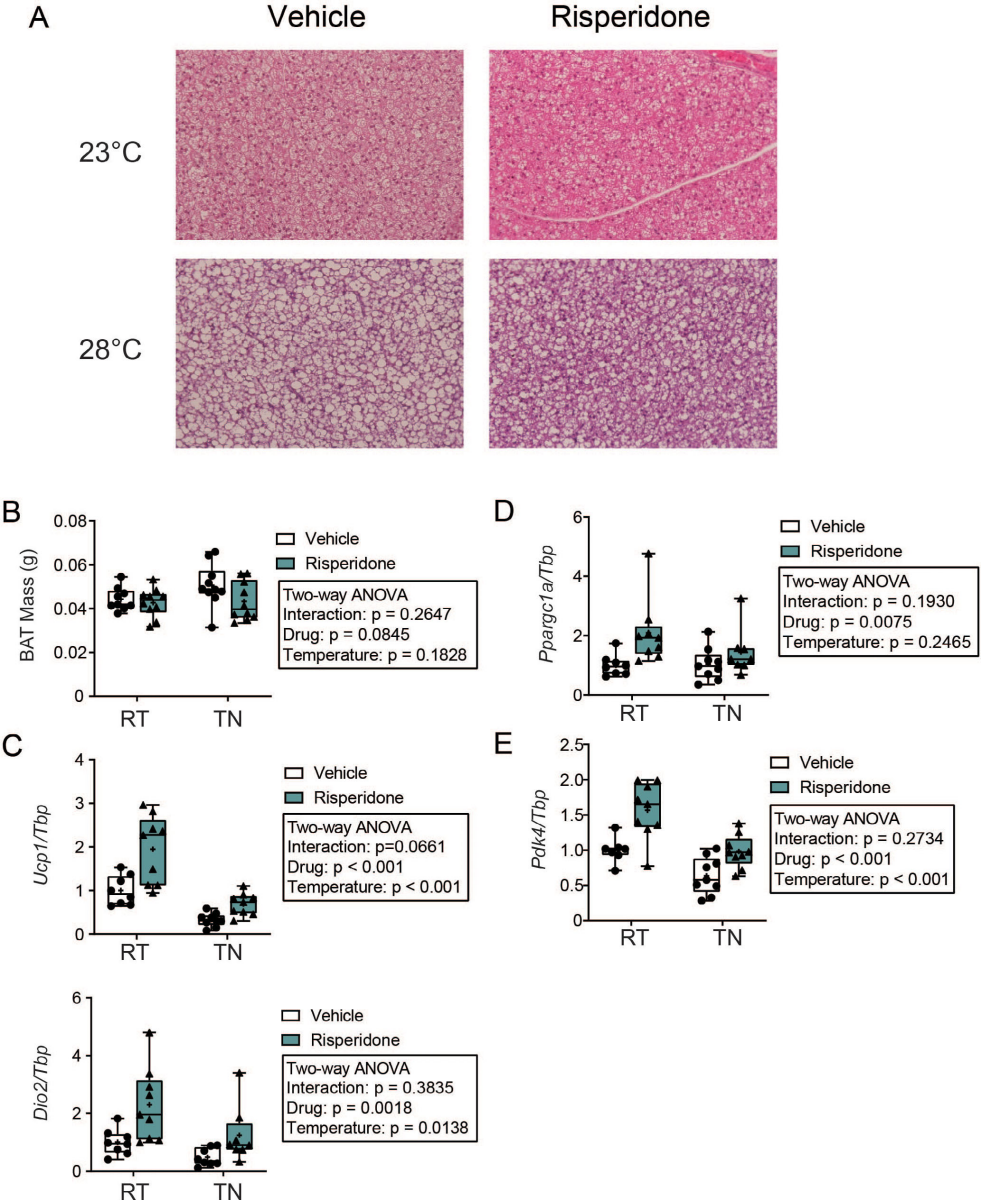
Risperidone‐induced thermogenesis in brown adipose tissue may be ameliorated by thermoneutral housing. (*A*) Representative images of H&E‐stained brown adipose tissue (BAT) imaged at 20×. (*B*) Endpoint mass of BAT, *n* = 9–10 per group. (*C*) Expression of genes involved in thermogenesis, *Ucp1* and *Dio2*, (*D*) mitochondrial biogenesis, *Ppargc1a*, and (*E*) adipocyte differentiation, *Pdk4* from BAT. Expression is normalized to *Tbp*. *n* = 7–9 per group. Plots represent median (horizontal line) ± 95% confidence interval (wide bars), minimum and maximum (T bars) values, and 2‐way ANOVA results with Tukey's multiple comparisons test performed only after a significant (*p* < 0.05) interaction term. + = arithmetic mean.

We next examined BAT mass, histology, and gene expression of thermogenic markers. Consistent with our previous work, H&E‐stained sections of BAT indicated reduced lipid droplet accumulation in BAT from RIS‐treated mice (Fig. 5*A*). {FIG5} As expected, lipid droplets were elevated in mice housed at thermoneutrality, yet RIS reduced lipid droplet size even at TN. RIS significantly increased *Ucp1, Ppargc1a, Pdk4*, and *Dio2* expression in BAT, and TN housing significantly suppressed all but *Ppargc1a* (Fig. 5*B*–*E*). Although no interaction terms reached statistical significance, TN housing tended to suppress the RIS‐induced upregulation of *Ucp1* (interaction *p* = 0.0661) (Fig. 5*C*).

**Fig 6:**
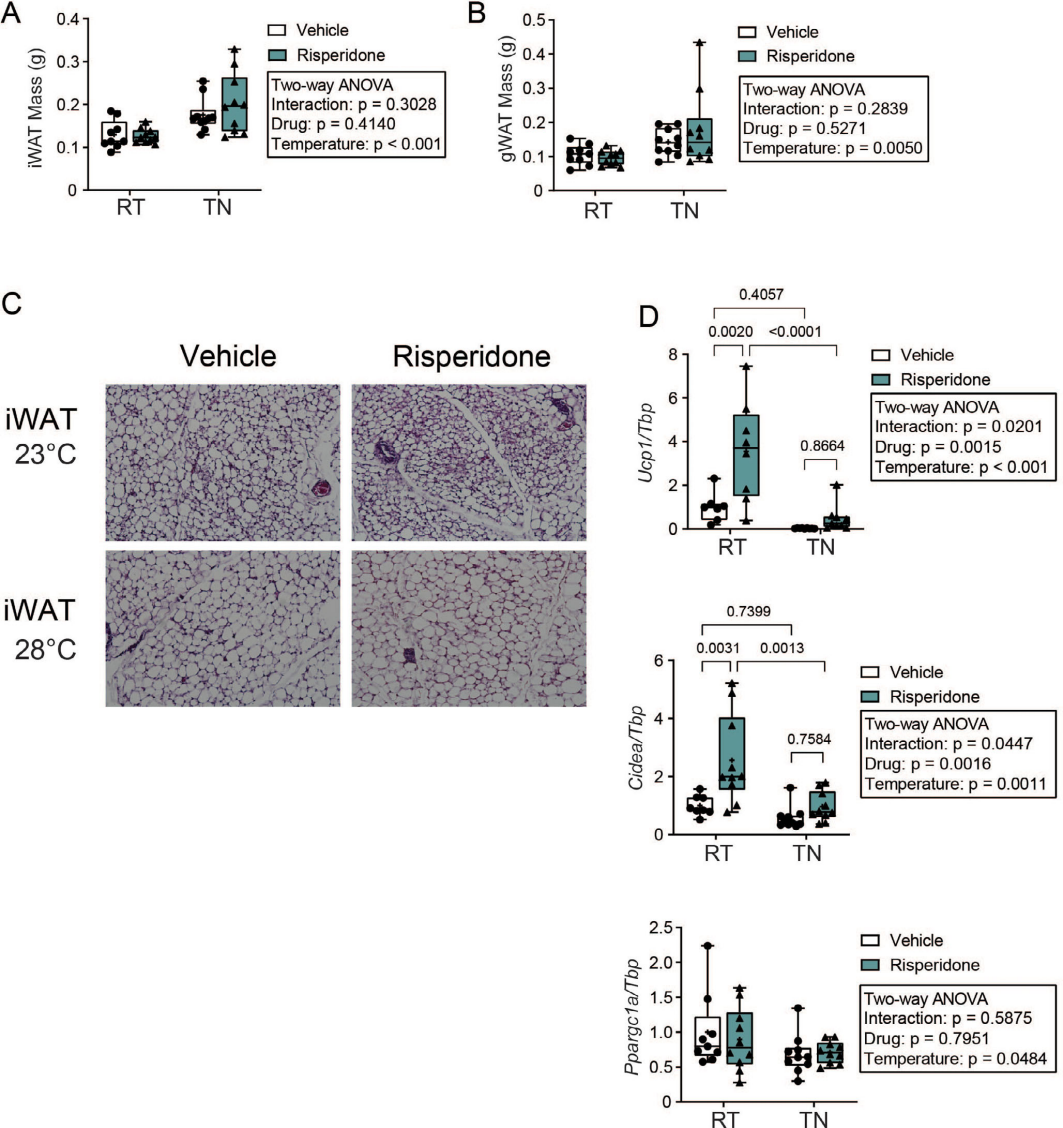
Thermoneutral housing prevents indices of risperidone‐induced beiging in inguinal white adipose tissue. (*A*) Endpoint mass of inguinal white adipose tissue (iWAT) and (*B*) gonadal WAT (gWAT), *n* = 9–10 per group. (*C*) Representative images of H&E‐stained iWAT imaged at 20×. (*D*) Expression of genes involved in beiging of WAT, *Ucp1* and *Cidea*, and (*E*) mitochondrial biogenesis, *Ppargc1a* normalized to *Tbp* expression, *n* = 7–9 per group. Plots represent median (horizontal line) ± 95% confidence interval (wide bars), minimum and maximum (T bars) values, and 2‐way ANOVA results with Tukey's multiple comparisons test performed only after a significant (*p* < 0.05) interaction term. + = arithmetic mean.

To address how white adipose tissue (WAT) was influenced by RIS and housing temperature, we weighed both the inguinal (iWAT) and gonadal (gWAT) adipose depots (Fig. 6*A*, *B*). {FIG6} Both depots increased in size in TN conditions, but weight was not affected by RIS treatment. However, iWAT, which is more likely to be beige than other depots, contained smaller adipocytes after treatment with RIS at RT (Fig. 6*C*). Interestingly and unlike what occurred in BAT, TN did suppress signs of beiging from RIS treatment (Fig. 6*C*). Consistent with this, gene expression of *Ucp1* and *Cidea* were significantly increased by RIS, and this was blocked by TN housing (interaction *p* < 0.05 for both genes) (Fig. 6*D*). However, the mitochondrial biogenesis marker *Ppargc1a* was only suppressed by TN and not altered by RIS (Fig. 6*D*). Taken together, these findings indicate that TN housing suppressed RIS‐induced thermogenesis.

## Discussion

Here we show that AA drug‐induced bone loss in mice is influenced by housing temperature in a compartment‐specific manner. After 4 weeks, RIS caused robust trabecular bone loss in the femur at RT. Although bone loss still occurred at TN, it was to a lesser extent. We also discovered the novel phenotype that RIS negatively impacts cortical bone acquisition promoted by TN housing temperature. Thus, it appears that AA drugs alone did not cause cortical bone loss; rather, RIS prevented thermoneutral‐induced cortical bone gain. Mice treated with RIS also displayed hallmarks of increased thermogenesis, including reduced lipid droplet size in BAT, and increased brown/beige adipocytes in iWAT with increased expression of thermogenic genes, which is achieved in part by increased SNS activity. These differences were greatest at RT and blunted at TN.

AA drugs are known to cause metabolic syndrome in humans. However, in the present study, as well as in our previous work, we have not observed an overt metabolic syndrome‐like phenotype in mice treated with risperidone (Supplemental Table S2; Fig. 6).^(^ ^20^ ^)^ We have also shown that 4 weeks of treatment does not affect insulin and glucose tolerance, while still causing bone loss.^(^ ^20^ ^)^ This is not surprising because risperidone has been shown to be more variable and milder in its metabolic side effects compared with olanzapine, which robustly increases weight and alters metabolism.^(^ ^24^, ^25^ ^)^ In humans, Meyer and colleagues found, retrospectively, that risperidone led to weight gain but did not increase triglycerides, glucose, or cholesterol to the extent of olanzapine, while Caro and colleagues found a 20% decrease in diabetic risk between risperidone and olanzapine.^(^ ^26^, ^27^ ^)^ Rodent studies have also shown variable effects of AA drugs, with dose and specific receptor pharmacology playing important roles in metabolic outcomes. In one study, Savoy and colleagues found that acute treatment with risperidone decreased plasma glucose levels and increased insulin in FVB/N mice.^(^ ^28^ ^)^ However, clamp studies have shown that a single dose of AA drugs in rats results in an immediate insulin resistance, but this varies depending on the specific drug.^(^ ^29^ ^)^ Dogs were also found to be impartial to overt metabolic syndrome from risperidone.^(^ ^30^ ^)^ There are few long‐term studies in animal models, but Ersland and colleagues demonstrated female Sprague Dawley rats continued to gain significant weight over 1 year of olanzapine treatment compared with vehicle.^(^ ^31^ ^)^ Taking the variable metabolic outcomes in humans and rodents into account, it is clear that overt metabolic syndrome‐like side effects are not required for bone loss and therefore not necessarily required for models of AA drug‐induced bone loss.

It has been well established that the SNS influences bone through epinephrine‐ and/or norepinephrine‐binding β2ARs expressed by osteoblasts, reducing osteoblast activity and increasing osteoclast activity via the production of RANKL.^(^ ^6^, ^7^, ^32^ ^)^ The mechanism of RIS‐induced bone changes is at least in part through elevated SNS activity. RIS‐induced bone loss can be prevented by both the non‐selective β‐blocker propranolol as well as global deletion of *Adrb2*, the gene encoding the β2AR.^(^ ^8^ ^)^ Notably, Beauchemin and colleagues found significant increases in c‐fos, a marker of neuronal activity, in the paraventricular nucleus (PVN) 2 and 4 hours post RIS, suggesting sympathetic nerve activation.^(^ ^10^ ^)^ Furthermore, treatment with the alpha‐adrenergic receptor antagonist yohimbine rescued metabolic outcomes of AA drugs.^(^ ^28^ ^)^ The mechanism of activation of the SNS by AA drugs is not entirely clear, although there is suggestive evidence that it may be through serotonin receptor inhibition, since AA drugs act as antagonists to serotonin receptors and serotonin receptor inhibition or downregulation in the hypothalamus has been shown to promote SNS activation.^(^ ^33^, ^34^ ^)^

We hypothesized that bone loss and thermogenesis from RIS were in part due to consequences of an unknown afferent signal for thermogenesis in rodents. Laboratory mice housed at room temperature are already in a sub‐thermoneutral state, in which they must utilize energy to maintain body temperature, and our previous studies have shown elevated energy expenditure immediately after RIS treatment, at a time when BAT markers were heightened.^(^ ^8^, ^10^ ^)^ We were successful in blocking evidence of beiging of WAT (Fig. 6), suggesting that at TN, no drive for thermogenesis existed that required development of thermogenic peripheral WAT. As such, the mechanism of RIS‐induced bone loss may in part be due to elevated drive for thermogenesis because bone loss was partly protected by TN housing. However, whether suppression of the SNS was responsible remains unclear. We did not determine circulating levels of epinephrine/norepinephrine post‐risperidone treatment at different housing temperatures. Indeed, McKie and colleagues recently demonstrated suppression of catecholamines with thermoneutral housing.^(^ ^35^ ^)^ These assays could support the hypothesis that the significant interactions we measured between drug and housing temperature on bone parameters and thermogenesis are SNS‐mediated. Nonetheless, additional bone loss in the cortical compartment, as well as elevated thermogenic markers in BAT, at thermoneutrality suggest a more direct, centrally mediated role for RIS in bone turnover and adipose tissue function.

There is evidence that BAT, iWAT, and bone share the same sympathetic neural circuit and thus could be equally altered by AA drugs.^(^ ^36^, ^37^, ^38^ ^)^ By retrograde tracing, Wee and colleagues demonstrated that separate administration of pseudorabies virus to tibia (red fluorescent protein [RFP] conjugated) and iWAT (green fluorescent protein [GFP] conjugated) colocalize through the spinal cord and into the brain.^(^ ^39^ ^)^ Of importance, they found RFP‐GFP‐positive neurons in the PVN of the hypothalamus, which as mentioned previously, is activated by RIS and is the origin of sympathetic stimuli.^(^ ^10^ ^)^ We found both WAT and BAT depots were significantly altered by AA drugs. This included expanding upon our previous finding of RIS‐induced *Ucp1* expression to other thermogenic genes, including *Cidea, Ppargc1α, Pdk4*, and *Dio2* in both BAT and iWAT.^(^ ^8^ ^)^ Our findings are similar to Cope and colleagues, who reported that RIS increased *Ucp1* expression and core body temperature.^(^ ^11^ ^)^ Other studies have shown either no effect of AA drugs on brown adipocytes or a suppression of differentiation in vitro, further supporting centrally mediated or indirect effects are more relevant.^(^ ^40^, ^41^ ^)^ In addition to being an indicator of SNS signaling, it is important to note that there is evidence that brown adipocytes directly affect bone, and thus our results of reduced BAT gene expression at TN could be more directly linked to the improved bone outcomes of both vehicle and AA drug‐treated mice. Continuing studies by Salisbury and colleagues have identified a unique population of UCP‐1‐positive brown‐like adipocytes within heterotopic bone lesions that were shown to support heterotopic ossification (HO) via maintenance of a hypoxic environment.^(^ ^42^ ^)^ Conversely, *Misty* mice have very low bone, in part due to limited BAT function causing SNS‐mediated compensation and beiging of WAT.^(^ ^43^ ^)^ Mechanisms of BAT signaling to other tissues, such as exosomal transport of miRNAs, have yet to be studied in a bone‐specific context and represent an interesting area for future research.

Because of evidence of dysregulated bone marrow adipose tissue (BMAT) with altered energy homeostasis, as well as the general tenet that osteoblasts and adipocytes share a common progenitor, we investigated if BMAT was altered in our study. Overall, only housing temperature altered BMAT (Fig. 3), and indices of osteoblast activity were elevated with RIS (Fig. 2), which does not support the hypothesis that induction of BMAT is a mechanism of AA‐induced bone loss. In contrast, previous work with RIS indicates that, if anything, BMAT is suppressed with RIS treatment, in a manner that is preventable by propranolol.^(^ ^8^ ^)^ Consistent with that, Turner and colleagues demonstrated BMAT responded to treatment with propranolol.^(^ ^16^ ^)^ Propranolol increased BMAT similar to TN housing, but when combined, mice were less able to gain marrow adiposity, suggesting overlapping mechanisms of increasing BMAT. In light of this, it is plausible that RIS would have a greater effect on BMAT in aged mice housed at RT where SNS activity appears to be more of an important contributor to BMAT loss.

In our past RIS‐treatment studies, we found that bone loss from risperidone was limited to the trabecular compartment.^(^ ^5^, ^8^ ^)^ Thus, we initiated experiments at 8 week of age, when trabecular bone reaches peak BV/TV.^(^ ^21^ ^)^ AA drugs are clinically prescribed to individuals with a large range of ages, including children and adolescents, and the elderly. Advanced aged mice, however, have very little quantifiable bone in the distal femur, which has prevented us from moving into aged models in the past. However, one surprising finding was that of a novel cortical phenotype in mice treated with RIS at thermoneutrality. We also recently showed that aged mice lose bone with cold stress.^(^ ^44^ ^)^ Together, these findings suggest that RIS would also cause cortical bone loss in aged mice under TN conditions. RIS did not cause cortical bone loss in our previous studies performed at standard RT.^(^ ^5^, ^8^, ^20^ ^)^ In general, Ct.Th and cross‐sectional area (Tt.Ar) of the femur are still increasing in female C57BL/6J mice up to 3 months of age, which is the endpoint of this study.^(^ ^21^ ^)^ Our results are consistent with the literature in showing that TN enhances this process.^(^ ^15^, ^17^ ^)^ In the case of RIS, endosteal bone acquisition at TN was inhibited, potentially through heightened resorption, since cortical *Tnfsf11* expression remained elevated in RIS‐treated mice at TN. Taken together, RIS appeared to alter the anabolic effect of TN housing temperature on cortical bone. Although the mechanism of bone gain at TN temperatures is not completely clear, there is evidence that it is at least in part through suppressed SNS activity because the β‐blocker propranolol partially protects against bone loss at RT.^(^ ^16^ ^)^ No compartment‐specific human studies have been performed with AA drugs, but it may be that the newly observed cortical phenotype at 28°C in mice better represents humans who are within thermoneutral range at RT. More studies will be needed to determine if the main contributor for increased fracture risk in patients taking AA drugs could be due to cortical rather than trabecular bone loss. Finally, it is not surprising that bone loss was not completely rescued by TN housing because of the evidence that AA drugs influence bone cell function directly. The efficacy of AA drugs to treat symptoms of mental disorders is due to their antagonizing effects on multiple receptors in the CNS, but it is important to note many of these receptors are also functionally expressed by bone cells.^(^ ^5^, ^45^, ^46^, ^47^, ^48^, ^49^ ^)^ We have confirmed via liquid chromatography with tandem mass spectrometry (LC‐MS/MS) measurable concentrations of RIS in marrow that peak 2 hours post gavage (max concentration 3120 nM), which is much higher than what is achieved in the serum during clinically relevant dosing.^(^ ^5^ ^)^ Studies from cell lines and primary osteoblasts and osteoclasts indicate that RIS may also have direct effects on bone. RIS suppresses MC3T3‐E1 cell osteoblast differentiation in a dose‐dependent manner.^(^ ^50^ ^)^ However, dopamine also suppresses osteoblast differentiation, suggesting RIS‐mediated effects may not be through dopamine receptor inhibition.^(^ ^5^ ^)^ Furthermore, RIS promotes osteoclast differentiation in vitro, which can also be blocked by dopamine.^(^ ^20^ ^)^ Apart from bone cells, there is evidence that AA drugs could modulate other cell types within the bone microenvironment to cause bone loss.^(^ ^51^, ^52^, ^53^, ^54^ ^)^ T cells aid in osteoclastogenesis and regulate bone turnover by secreting cytokines that affect RANKL‐RANK signaling.^(^ ^55^, ^56^, ^57^ ^)^ A recent study by May and colleagues demonstrated RIS suppresses the immune system, with reductions in cytokine levels and an inability to mount an immune response to a vaccine, yet it remains to be investigated how this would lead to elevated RANKL and altered bone turnover.^(^ ^53^, ^58^ ^)^

Our study is the first to assess how environmental temperature influences the effects of AA drugs in mice. Despite a link between environmental temperature and SNS activation to maintain body temperature, we found that the AA drug RIS still impairs cortical bone acquisition at TN but that the trabecular bone phenotype was a significant improvement from the bone loss observed at RT. Expansion upon these studies with additional pharmacologic and genetic models would be needed to support the mechanism of TN housing suppressing SNS activity. Namely, previous studies have shown that the β‐blocker propranolol attenuates risperidone‐induced bone loss, but it is unclear if it would also attenuate the cortical bone loss observed in this study at TN.^(^ ^8^ ^)^ Another limitation of this study is that we only included female mice, which were previously shown to have SNS‐mediated bone loss, and expanding to males mice, and mice of different ages, would expand the translatability of these findings to humans.^(^ ^8^ ^)^ Although microneurographic experiments would be needed to directly test the role of SNS tone, future studies would need to include catecholamine measurements to determine if marrow and serum levels are attenuated by TN housing.

Collectively, these findings further support the notion that AA drug‐induced bone loss is multifactorial, with direct, CNS‐mediated, and endocrine/paracrine effects from other tissues being likely contributors. Although not addressing bone, Eng and colleagues found statistically significant and different responses to cancer treatment when mice were housed at RT and TN based on SNS signaling via the β2AR.^(^ ^59^ ^)^ Therefore, preclinical studies for drug treatments, especially those that affect the CNS/SNS, should consider housing temperature during the experimental design phase. Although we did not directly measure core body temperature after AA drug treatment, it should also be noted that risks of hypothermia (reduced core body temperature, decreased sympathetic output) and hyperthermia (increased core body temperature, increased sympathetic output) from patients taking AA drugs are documented, further stressing the need for preclinical studies to be designed with rodent housing temperature in mind.^(^ ^60^, ^61^ ^)^

## Disclosures

All authors state that they have no conflicts of interest.

### Peer Review

The peer review history for this article is available at https://publons.com/publon/10.1002/jbm4.10541.

## Supporting information


**Appendix S1.** Supporting Information.
