# Supplementary material for: Housing Temperature Influences Atypical Antipsychotic Drug‐Induced Bone Loss in Female C57BL/6J Mice
Source: JBMR Plus. 2021 Sep 7;5(10):e10541. doi: 10.1002/jbm4.10541 (PMC8520062; doi:10.1002/jbm4.10541)
Supplement: Supplementary file 1 — Appendix S1. Supporting Information. [file JBM4-5-e10541-s001.docx]

Table S1. RT-PCR primers.

| **Gene** | **Forward primer** | **Reverse primer** | **Source** |
| --- | --- | --- | --- |
| *5ht2a* | 5'-TAT GCT GCT GGG TTT CCT TGT C-3' | 5'-CAC AAA AGA GCC TAT GAG AAC A-3' | IDT |
| *Acp5* | 5’-AAT GCC TCG ACC TGG GA-3’ | 5'-CGT AGT CCT CCT TGG CTG CT-3' | IDT^(1)^ |
| *Cidea* | 5'-TGC TCT TCT GTA TCG CCC AGT -3' | 5'-GCC GTG TTA AGG AAT CTG CTG -3' | IDT^(2)^ |
| *Ctsk* | 5'-GCA GAG GTG TGT ACT ATG -3' | 5'-GCA GGC GTT GTT CTT ATT-3' | IDT |
| *Dio2* | 5'-CAG TGT GGT GCA CGT CTC CAA TC-3' | 5'-TGA ACC AAA GTT GAC CAC CAG-3' | IDT^(3)^ |
| *Fabp4* | 5′-GCG TGG AAT TCG ATG AAA TCA-3′ | 5′-CCC GCC ATC TAG GGT TAT GA-3′ | IDT^(4)^ |
| *Tnfrsf11b* | 5'-GAA GAA GAT CAT CCA AGA CAT TGA C-3' | 5'-TCC ATA AAC TGA GTA GCT TCA GGA G-3' | IDT^(5)^ |
| *Bglap* | 5'-ACG GTA TCA CTA TTT AGG ACC TGT-3' | 5'-ACT TTA TTT TGG AGC TGC TGT GAC-3' | IDT^(6)^ |
| *Pdk4* | 5'-AAA GTG GGT CTG TGG CAT TG-3' | 5'-AAG GTT TGT ACT CGT GTT TGT G-3' | Primer Design |
| *Ppargc1a* | 5'-TGA TGT GAA TGA CTT GGA TAC AGA CA-3' | 5'-GCT CAT TGT TGT ACT GGT TGG ATA TG-3' | IDT^(3)^ |
| *Pparg2* | 5'-GCA TGG TGC CTT CGC TGA-3' | 5'-TGG CAT CTC TGT GTC AAC CAT G-3' | IDT^(3)^ |
| *Tnfsf11* | 5'-TTT GCA CAC CTC ACC ATC AAT-3' | 5'-CCC TTA GTT TTC CGT TGC TTA AC-3' | Primer Design |
| *Runx2* | 5'-GAC AGA AGC TTG ATG ACT CTA AAC C-3' | 5'-TCT GTA ATC TGA CTC TGT CCT TGT G-3' | IDT^(7)^ |
| *Ucp1* | 5'-ACT GCC ACA CCT CCA GTC ATT-3' | 5'-CTT TGC CTC ACT CAG GAT TGG-3' | IDT^(3)^ |
| *Hprt* | 5′-AAG CCT AAG ATG AGC GCA AG-3 | 5′-TTA CTA GGC AGA TGG CCA CA-3′ | IDT^(8)^ |
| *Tbp1* | 5'-GAA GCT GCG GTA CAA TTC CAG -3' | 5'-CCC CTT GTA CCC TTC ACC AAT -3' | IDT^(9)^ |

Table S2. Body composition of female mice treated with risperidone or vehicle, at room temperature or thermoneutrality, for four weeks.

| DXA-body composition |  |  | **Room Temperature** | | | **Thermoneutral** | | **2-Way ANOVA *p*-value** | | |
| --- | --- | --- | --- | --- | --- | --- | --- | --- | --- | --- |
|  |  |  | **Veh (n=9)** | **RIS (n=10)** | **Veh (n=10)** | | **RIS (n=10)** | **Drug** | **Temp.** | **Int.** |
| BL Body mass (g) |  |  | 18.9 ± 0.3 | 18.5 ± 0.2 | 19.2 ± 0.4 | | 19.8 ± 0.6 |  |  |  |
| End Body mass (g) |  | | 19.7 ± 0.3 | 20.7 ± 0.3 | 19.9 ± 0.3 | | 21.4 ± 0.7 | **0.0079** | 0.3290 | 0.5301 |
| *Body mass change from baseline (%)* |  |  | 4.5 ± 1.2 | 11.7 ± 0.9 | 4.0 ± 1.1 | | 8.3 ± 1.1 | **<0.0001** | 0.0871 | 0.2032 |
| BL Lean mass (g) |  |  | 14.6 ± 0.2 | 14.5 ± 0.2 | 15.5 ± 0.3 | | 15.8 ± 0.4 |  |  |  |
| End Lean mass (g) |  |  | 15.9 ± 0.2 | 16.6 ± 0.2 | 15.5 ± 0.3 | | 16.6 ± 0.4 | **0.0048** | 0.4363 | 0.5616 |
| Lean Mass/Body Mass (%) |  |  | 86.1 ± 0.8 | 85.7 ± 0.7 | 83.6 ± 1.0 | | 82.6 ± 1.0 | 0.4392 | **0.0041** | 0.7391 |
| *Lean mass change from baseline (%)* |  |  | 8.6 ± 1.2 | 15.0 ± 1.4 | -0.7 ± 0.8 | | 5.1 ± 1.4 | **<0.0001** | **<0.0001** | 0.6541 |
| BL Fat mass (g) |  |  | 3.0 ± 0.2 | 2.9 ± 0.2 | 2.5 ± 0.1 | | 2.9 ± 0.2 |  |  |  |
| End Fat mass (g) |  |  | 2.6 ± 0.2 | 2.8 ± 0.2 | 3.0 ± 0.2 | | 3.5 ± 0.3 | 0.1015 | **0.0125** | 0.5332 |
| Fat Mass/Body Mass (%) |  |  | 14.1 ± 0.9 | 14.5 ± 0.7 | 16.3 ± 0.9 | | 17.3 ± 1.0 | 0.4318 | **0.0074** | 0.6981 |
| *Fat mass change from baseline (%)* |  |  | -14.5 ± 6.0 | -3.2 ± 4.8 | 20.3 ± 8.9 | | 23.2 ± 7.5 | 0.3099 | **<0.0001** | 0.5435 |

Data presented as mean ± SEM.

*Abbreviations:* DXA, dual energy X-ray absorptiometry; BL, baseline; End, endpoint; Veh, vehicle; RIS, risperidone; Temp., temperature; Int., interaction.

Table S3. Areal bone parameters in female mice treated with risperidone or vehicle, at room temperature or thermoneutrality, for four weeks.

| DXA-Bone |  | **Room temperature** | | **Thermoneutral** | | **2-Way ANOVA *p*-value** | | |
| --- | --- | --- | --- | --- | --- | --- | --- | --- |
|  |  | **Veh (n=9)** | **RIS (n=10)** | **Veh (n=10)** | **RIS (n=10)** | **Drug** | **Temp.** | **Int.** |
| BL Total aBMD (mg/cm^2^) |  | 455 ± 3 | 448 ± 3 | 465 ± 5 | 455 ± 4 |  |  |  |
| End Total aBMD (mg/cm^2^) |  | 480 ± 5 | 481 ± 4 | 493 ± 4 | 490 ± 5 | 0.8404 | **0.0172** | 0.6108 |
| *aBMD change from BL (%)* |  | 5.5 ± 0.8 | 7.4 ± 0.8 | 6.1 ± 1.4 | 7.6 ± 0.8 | 0.0890 | 0.7102 | 0.7924 |
| BL Total aBMC (mg/cm^2^) |  | 321 ± 10 | 318 ± 10 | 338 ± 10 | 342 ± 10 |  |  |  |
| End Total aBMC (mg/cm^2^) |  | 376 ± 8 | 371 ± 7 | 401 ± 8 | 395 ± 10 | 0.5292 | **0.0057** | 0.9995 |
| *aBMC change from BL (%)* |  | 17.1 ± 1.4 | 16.9 ± 1.6 | 18.7 ± 1.2 | 15.7 ± 0.9 | 0.2219 | 0.8951 | 0.2954 |
| BL Femur aBMD (mg/cm^2^) |  | 51 ± 1 | 50 ± 1 | 52 ± 1 | 52 ± 1 |  |  |  |
| End Femur aBMD (mg/cm^2^) |  | 57 ± 1 | 59 ± 1 | 60 ± 1 | 62 ± 1 | 0.0941 | **0.0028** | 0.7905 |
| *Femur aBMD change from BL (%)* |  | 13.7 ± 2.5 | 17.3 ± 2.2 | 14.3 ± 1.8 | 19.2 ± 1.5 | 0.2167 | 0.0744 | 0.6315 |

Data presented as mean ± SEM.

*Abbreviations:* DXA, dual energy X-ray absorptiometry; aBMD, areal bone mineral density; aBMC, areal bone mineral content; BL, baseline; End, endpoint; Veh, vehicle; RIS, risperidone; Temp., temperature; Int., interaction.

**Supplemental References**

1. Wiren KM, Zhang XW, Toombs AR, Kasparcova V, Gentile MA, Harada SI, Jepsen KJ. Targeted overexpression of androgen receptor in osteoblasts: Unexpected complex bone phenotype in growing animals. Endocrinology. 2004;

2. Seale et al. PRDM16 Controls a Brown Fat/Skeletal Muscle Switch. Bone. 2008;23(1):1–7.

3. Cooper MP, Uldry M, Kajimura S, Arany Z, Spiegelman BM. Modulation of PGC-1 coactivator pathways in brown fat differentiation through LRP130. J. Biol. Chem. 2008;

4. Li J, Takaishi K, Cook W, McCorkle SK, Unger RH. Insig-1 “brakes” lipogenesis in adipocytes and inhibits differentiation of preadipocytes. Proc. Natl. Acad. Sci. U. S. A. 2003;

5. Irwin R, Lee T, Young VB, Parameswaran N, McCabe LR. Colitis-induced bone loss is gender dependent and associated with increased inflammation. Inflamm Bowel Dis [Internet]. 2013;19(8):1586–97. Available from: http://www.ncbi.nlm.nih.gov/pubmed/23702805

6. Ontiveros C, McCabe LR. Simulated microgravity suppresses osteoblast phenotype, runx2 levels and AP-1 transactivation. J. Cell. Biochem. 2003;

7. Ontiveros C, Irwin R, Wiseman RW, McCabe LR. Hypoxia suppresses runx2 independent of modeled microgravity. J Cell Physiol [Internet]. 2004;200(2):169–76. Available from: http://www.ncbi.nlm.nih.gov/pubmed/15174088

8. Vengellur A, LaPres JJ. The role of hypoxia inducible factor 1α in cobalt chloride induced cell death in mouse embryonic fibroblasts. Toxicol. Sci. 2004;

9. Sanchez-Gurmaches ; Guertin D. Adipocytes arise from multiple lineages that are heterogeneously and dynamically distributed Access. Nat Commun. 2014;5(1):4099.
